# Combined CT radiomics of primary tumor and metastatic lymph nodes improves prediction of loco-regional control in head and neck cancer

**DOI:** 10.1038/s41598-019-51599-7

**Published:** 2019-10-23

**Authors:** Marta Bogowicz, Stephanie Tanadini-Lang, Matthias Guckenberger, Oliver Riesterer

**Affiliations:** Department of Radiation Oncology, University Hospital Zurich, University of Zurich, Zurich, Switzerland

**Keywords:** Prognostic markers, Cancer imaging, Head and neck cancer, Tumour heterogeneity

## Abstract

Loco-regional control (LRC) is a major clinical endpoint after definitive radiochemotherapy (RCT) of head and neck cancer (HNC). Radiomics has been shown a promising biomarker in cancer research, however closer related to primary tumor control than composite endpoints. Radiomics studies often focus on the analysis of primary tumor (PT). We hypothesize that the combination of PT and lymph nodes (LN) radiomics better predicts LRC in HNC treated with RCT. Radiomics analysis was performed in CT images of 128 patients using Z-Rad implementation (training n = 77, validation n = 51). 285 features were extracted from PT and involved LN. Features were preselected with the maximum relevance minimum redundancy method and the multivariate Cox model was trained using least absolute shrinkage and selection operator. The mixed model was based on the combination of PT and LN radiomics, whereas the PT model included only the PT features. The mixed model showed significantly higher performance than the PT model (p < 0.01), c-index of 0.67 and 0.63, respectively; and better risk group stratification. The clinical nodal status was not a significant predictor in the combination with PT radiomics. This study shows that the LRC can be better predicted by expansion of radiomics analysis with LN features.

## Introduction

Head and neck cancer (HNC) accounts for 5% to 10% of new cancer cases in developed countries and the incidence of cases related to human papilloma virus (HPV) infection is increasing^1–3^. Standard of care for treatment of advanced or inoperable cases is radiochemotherapy (RCT). Due to the heterogeneous nature of this disease, the cure rates at 5 years after RCT vary from 50% to 80%^4,5^. In the era of precision medicine, various prognostic biomarkers, such as gene mutation, serum biomarkers and tumor hypoxia have been proposed to improve patient stratification and to consequently increase treatment response by treatment intensification in bad-performing subgroups and reduce side effects by treatment deintensification in well-performing subgroups^6–8^.

In recent years radiomics has been proposed as a novel technique for identification of prognostic imaging biomarkers^9,10^. It is based on a big data approach to quantitative image analysis, where hundreds of features are extracted from a region of interest (ROI) and are correlated with clinical outcomes. These features comprise information about the ROI shape, intensity distribution, texture and heterogeneity. Radiomic signatures were shown to be promising prognostic biomarkers for different endpoints, however its efficiency varies depending on the complexity of an endpoint. Starting from endpoints, which are directly linked to tumor biology, such as HPV status, hypoxia or Gleason score, where the accuracy of prediction was good (area under receiver operating characteristics > 0.8)^11–14^. Similarly, studies correlating radiomic features with local tumor control showed good discrimination with concordance indexes (c-index) above 0.7^11,15,16^. On the other hand, overall survival, a highly relevant clinical endpoint, is influenced not only by tumor subtype or treatment but also by patient performance status and lifestyle. Therefore radiomics studies investigating overall survival as an endpoint showed lower discriminatory performance with a c-index around 0.65^17,18^.

Especially in the case of HNC, loco-regional tumor control (LRC) combining control of primary tumor and lymph node metastases is a very important composite clinical endpoint, because it correlates with overall survival, and often is the primary endpoint in clinical trials investigating the effect of RCT. In this study we hypothesize that the full potential of radiomics analysis has not yet been explored for the analysis of the intermediate endpoint loco-regional control (LRC). Previously, only one study tried to correlate primary tumor radiomic features to LRC with unsatisfactory results in the case of CT imaging^19^. In the radiomics-based treatment response modelling the evaluated ROI has usually been limited to the primary tumor. In addition to this ‘traditional’ analysis of the primary tumor region we hypothesized, that additional analysis of the macroscopically involved lymph nodes will improve outcome modelling. Further, we investigated new features describing the distribution of lymph nodes around the primary tumor. To show the added value of lymph nodes radiomics, for the prediction of LRC, we compared it to the models based on primary tumor and N stage as well as to the model predicting local tumor control.

## Material and Methods

### Studied population and image acquisition

Head and neck squamous cell carcinoma patients, stage III/IV with N+ disease, were retrospectively enrolled in this study, which was approved by the Swissethics. The training cohort comprised 77 patients, for which data was collected retrospectively, whereas the validation cohort of 51 patients was a part of an institutional phase II prospective study (NCT01435252). This study was carried out in accordance with Swissethics guidelines and regulations. Patients in the retrospective cohort gave informed general consent and informed specific consent was obtained from the prospective cohort. Exclusion criteria were induction chemotherapy, prior surgery (biopsy allowed) or planned neck dissection after completion of RCT. All patients were treated with definitive RCT with a radiation dose of 70 Gy. The chemotherapy differed between the cohorts. In the training cohort, cisplatin (40 mg/m^2^, up to 7 cycles) or cetuximab (loading dose 400 mg/m^2^ followed by 250 mg/m^2^ weekly) was given. The validation cohort was treated in the study protocol with concurrent weekly cisplatin/cetuximab (same doses as in training cohort) with or without consolidation cetuximab (500 mg/m^2^ biweekly × 6). Loco-regional failure was confirmed by a 18F-FDG PET scan and a biopsy (see details in Table 1).

**Table 1:** Studied cohorts details and CT acquisition parameters.

	Training cohort	Validation cohort
Number of patients	77	51
Local control failures	18 (23%)	13 (26%)
Loco-regional control failures	28 (36%)	17 (33%)
Median follow-up [months]	54	26
N stage	N1 = 5 N2a = 1 N2b = 38 N2c = 28 N3 = 5	N1 = 5 N2a = 2 N2b = 28 N2c = 14 N3 = 2
CT scanner	Siemens SOMATOM Definition AS (n = 18)	Siemens SOMATOM Definition AS (n = 51)
	Siemens SOMATOM Volume Zoom (n = 35)	
	Siemens SOMATOM PLUS 4 (n = 17)	
	GE Discovery (n = 7)	
Slice thickness [mm]	2.00–3.27	2.00
In-plane resolution [mm]	0.98 (0.84–1.56)	0.98 (0.98–1.56)
kV	120; 140	120
mAs	214 (60–450)	450 (183–450)

Patients underwent a contrast-enhanced planning CT according to an institutional protocol. Details on the scanning protocol are shown in Table 1. The training cohort showed heterogeneity in acquisition parameters due to its retrospective nature, whereas the validation cohort was scanned with the standardized CT settings.

### Radiomics analysis and lymph nodes distribution analysis

Radiomic features were extracted separately from the primary tumor (PT) region and the involved lymph nodes (LN) detected by FDG-PET and confirmed by fine needle biopsy. Only the LN receiving full radiation dose of 70 Gy were included in the analysis. If more than one LN was present, all LN were included in one ROI. The original radiotherapy contours were adapted for the presence of artifacts. Contours were removed from the artifacts-affected slices and only patients with less than 50% of artifacts volume in the region of interest were eligible.

All images were resampled to 3.3 mm cubic voxels, the largest voxel dimension in the available dataset. The Hounsfield Unit range of −20 to 180 was used to limit the analysis to soft tissue. Z-Rad radiomics software implementation was used to extract 285 radiomic features: shape (n = 18), intensity (n = 17), texture (n = 72), wavelet (n = 178). Only the low-pass (LLL) and high-pass (HHH) filtered maps were analyzed. Definition of the extracted features can be found in the Imaging Biomarker Standardization Initiative report^20^. Details on the calculation of wavelet and shape features were previously described in the supplementary materials^21^.

Lymph node distribution in respect to primary tumor location (LNPT) was additionally quantified with 14 new features (see supplement for more details):

- distribution of distances between primary tumor and lymph nodes based on the center of the mass (n = 5),
- distribution of distances between primary tumor and lymph nodes weighted by lymph node’s volume (n = 2),
- distribution of distances between primary tumor and lymph nodes weighted by lymph node’s volume and normalized by tumor volume (n = 2),
- distribution of the smallest distances between the analyzed structures based on Kruskal algorithm (n = 2),
- number of clusters based on Calinski-Harabasz index and all points within lymph nodes contour (n = 1),
- principal component analysis of all the points within primary tumor and lymph nodes to define elongation and flatness (n = 2).

### Prediction of loco-regional control

**Figure 1:**
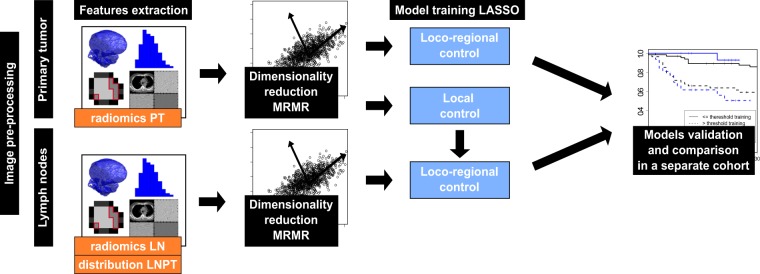
Scheme of the analysis. Radiomic features were extracted from primary tumor (PT) and lymph nodes metastases (LN) as well as from the distribution of LN around PT (LNPT). These data were used to predict loco-regional control. Performance of the models based on PT only and combination of PT and LN was compared in a separate validation cohort.

Two model types were trained to predict LRC using radiomic features. One based solely on PT radiomic features and the second based on PT features, LN features as well as LNPT features (Fig. 1). The minimum redundancy maximum relevance (MRMR) method was used to preselect features. The feature count for MRMR was defined in the principal component analysis, as the number of principal components, which explains 95% of data variance. The MRMR was repeated 1000 times with randomly selected samples using the bootstrap procedure. The redundancy between the features was defined by the Spearman correlation. Features, which achieved at least 60% selection rate, were included in the final set. The MRMR selection was performed separately for PT, LN and LNPT features. The final classification was performed with least absolute shrinkage and selection operator LASSO (100 times bootstrap, 5-fold cross-validated). Features, which had at least 60% selection rate, were chosen for the final model.

In the first model type (PTmodel), the PT features were directly linked to LRC. In the second model type (mixed model), PT radiomic features were linked to local tumor control (LC rad score) and the results were used for LRC modelling together with a) LN and b) LNPT features. For comparison, a model based on the PT radiomics and N stage was trained. The performance of the models was tested in the validation cohort using the Wilcoxon test (p < 0.05) and the bootstrap method with 100 randomly selected samples to calculate the concordance index (c-index) distribution. Additionally, patients were split into two risk groups based on the threshold maximizing sensitivity and specificity at 18 months.

## Results

The MRMR showed 9 non-redundant PT radiomic features linked to LC. The multivariate Cox model consisted of 3 features (LC rad score) and achieved good discriminatory performance on the validation set (c-index 0.70). For comparison, the LRC model comprised 3 PT radiomic features out of 10 selected in MRMR. It showed significantly worse performance than the LC model (Table 2). Two out of three features in these models were the same.

**Table 2:** Performance of the local (LC) and loco-regional (LRC) control prediction models depending on the input. Combination of PT and LN radiomics improved prediction of LRC (p-value < 0.05). LC rad score: prediction of LC based on the PT radiomics.

model input	endpoint	features	c-index 5-fold CV training	c-index validation (95% CI)
PT radiomics	LC	GLSZM zone entropy LLL NGTDM complexity LLL GLCM entropy	0.81	0.70 (0.68–0.71)
PT radiomics	LRC	NGTDM complexity LLL NGTDM complexity LLL GLCM entropy	0.67	0.63 (0.62–0.64)
LN radiomics	LRC	thickness SD spherical disproportion major axis histogram kurtosis	0.72	0.60 (0.58–0.61)
PT + LN radiomics	LRC	LC rad score thickness SD spherical disproportion major axis histogram kurtosis	0.75	0.67 (0.66–0.68)
PT radiomics distribution LNPT	LRC	Distribution LNPT feature not significant in the multivariate model	—	—
PT radiomics N stage	LRC	N stage not significant in the multivariate model	—	—

Three combined models for LRC prediction were investigated: the LC prediction using PT radiomics (LC rad score) was used as an input prediction of LRC together with a) LN radiomics, b) LNPT radiomics and c) N stage. The training of two models was unsuccessful as LNPT radiomic features and N stage were discarded in the LASSO selection. However, four LN radiomic features were selected in combination with LC rad score (mixed model) resulting in improved prediction of LRC (c-index = 0.67). This model was also significantly better than the model based solely on LN radiomics p-value < 0.05 (Table 2).

**Figure 2:**
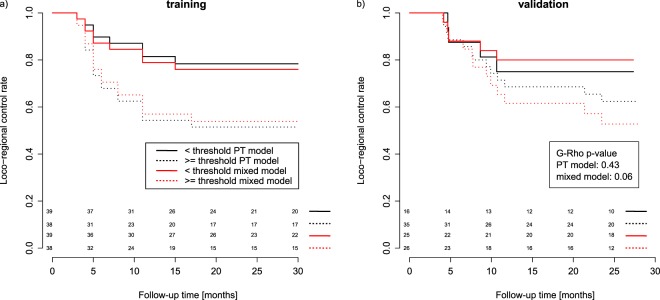
The loco-regional control rate for risk groups based on the PT model and the mixed PT + LN model (**a**) training cohort, (**b**) validation cohort. The mixed model showed better patient stratification in the validation cohort.

**Figure 3:**
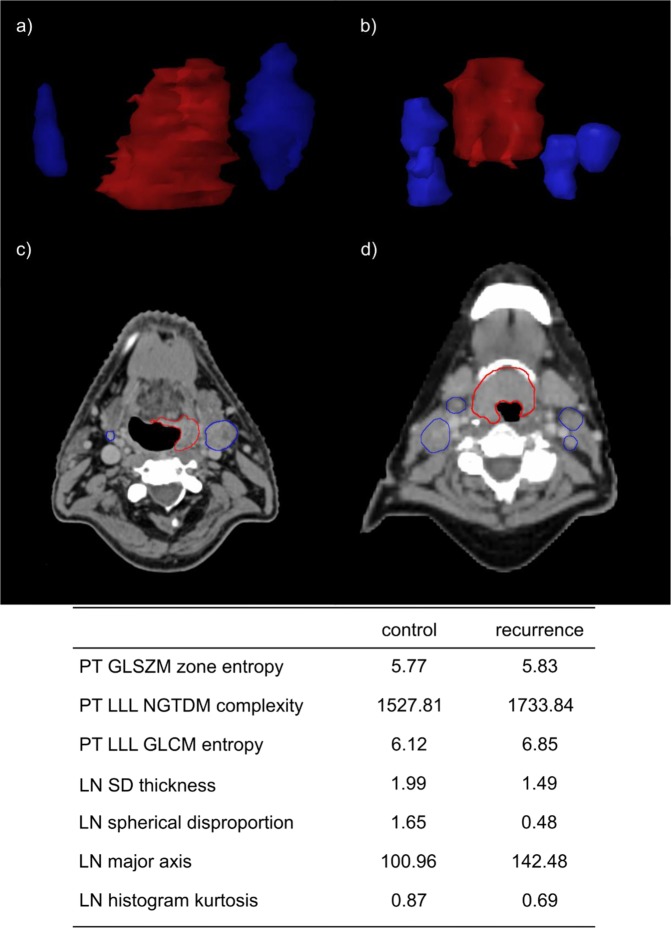
Example of CT images for patient with loco-regional control (**a**,**c**) and without (**b**,**d**). The images a and b show distribution of lymph nodes (blue) around primary tumor (red). The images c and d show representative CT slices. Additionally, radiomic features from the combined model for those two patients are presented in the table below.

The mixed model also allowed for a better risk group stratification in comparison to the PT model (Fig. 2). The PT threshold failed to stratify patients in the validation cohort, whereas the mixed model threshold was close to significant (p-value = 0.06). An exemplary mixed model radiomic features together with respective CT images and contours are presented in Fig. 3 for two patients, with and without loco-regional control.

## Discussion

Cancer related prognostic studies based on radiomics so far have been limited to analysis of the primary tumor only. However, in some tumor types such as head and neck cancer, control of the primary tumor and lymph node metastases is prerequisite for cancer cure. In addition, studies investigating the semantic CT phenotype of lymph nodes, suggested its close link with a specific tumor biology. The extracapsular spread and cystic metastases were shown to correlate with HPV infection^22,23^. This study aimed at improved prediction of loco-regional control after definitive radiochemotherapy by exploration of quantitative imaging features from macroscopically involved lymph nodes (LN). Additionally, 14 new features were defined to describe spatial distribution of lymph nodes in relation to the primary tumor (PT). An improved discriminatory power was observed for a mixed model (combination of radiomic features from PT and LN) in comparison to the PT model, c-index of 0.67 and 0.63, respectively. The mixed model was also better for patient risk group stratification on the level of the validation cohort. Interestingly, the distribution of the LN around the PT as well as the N stage, were not significant predictors when combined with PT radiomics.

Three out of four selected LN features belonged to the shape subgroup. All metastatic lymph nodes were analyzed as one ROI and thus the interpretation of the shape features is not the same as for the primary tumor. For example, spherical disproportion should be rather interpreted as a “spread of disease” parameter than as complexity of lymph node shape. Larger lymph node spread (larger major axis and larger spherical disproportion) indicated worse prognosis. Similarly, larger thickness SD was linked to worse prognosis, meaning larger variability in LN diameters is a negative prognostic factor. Despite the fact that most of the selected LN features correspond to geometry, the location of LN in relation to PT did not seem to influence LRC, as neither the N stage nor the distribution features (LNPT) were significant in multivariate modelling when combined with PT features. On the level of the primary tumor, similar features were selected for prediction of LC and LRC, indicating that selection of PT features was biased towards the local tumor control events and explaining lower performance of the LRC model in comparison to LC model.

Primary tumor radiomics has been previously evaluated for LRC prediction in HNC by Vallieres *et al*.^19^. They found that PET radiomic features were linked with LRC, whereas CT radiomic features were not prognostic, which contradicts our results. However, they did not report any artifact postprocessing steps, which could explain the contradictory results^24^. Carvalho *et al*.^25^ observed improved prediction of overall survival in lung cancer when lymph node PET radiomic features were incorporated. However, the performance of obtained models was generally low, c-index 0.56–0.59. As a next step, the added value of lymph node radiomic features should be tested against clinical factors other than N stage. Finally, primary tumor radiomic features, lymph node radiomic features and clinical prognostic factors should be incorporated into multifactorial model to predict overall survival.

This is a preliminary study to show the potential of lymph node radiomic features as a complementary prognostic factor in LRC modelling in HNC. These results need to be further validated in a larger, multicenter cohort. Certain technical aspects should also be investigated. The analysis was performed on contrast-enhanced CT images. However, the contrast protocol was not standardized in the studied cohorts. At the current stage, little is known about the impact of contrast protocol (concentration and scanning delay) on the stability of radiomic features^26^. Metal artifacts are frequent in the head and neck region. In this study, contours from slices with artifacts were removed. This method has been tested for primary tumor, but not for lymph nodes^24^. Further validation of these results in the images reconstructed with iterative metal artifact reduction algorithms would be of interest. Similarly, other radiomics robustness studies for regions of interest encompassing lymph nodes are missing, e.g. inter-observer delineation variability^27^.

In conclusion, we showed that expanding radiomics analysis with LN radiomic features improves model performance for prediction of the more complex and clinically more relevant endpoint LRC. These results should be further validated for other cohorts and investigated for other tumor types, where control of lymph node metastases in addition to primary tumor control impacts on patient cure.

## Supplementary information

Supplementary material
