# Supplementary material for: Combined CT radiomics of primary tumor and metastatic lymph nodes improves prediction of loco-regional control in head and neck cancer
Source: Sci Rep. 2019 Oct 23;9:15198. doi: 10.1038/s41598-019-51599-7 (PMC6811564; doi:10.1038/s41598-019-51599-7)
Supplement: Supplementary file 1 — Supplementary material [file 41598_2019_51599_MOESM1_ESM.pdf]

Supplement: Combined CT radiomics of primary tumor and metastatic lymph nodes improves prediction of loco-regional control in head and neck cancer

Marta Bogowicz (1), Stephanie Tanadini-Lang (1), Matthias Guckenberger (1), Oliver Riesterer (1)

(1) Department of Radiation Oncology, University Hospital Zurich, University of Zurich, Zurich, Switzerland

Distribution features

The distribution features aim at quantification of the spread of regional metastatic disease (metastatic lymph nodes LN) around the primary tumor (PT). This information is based purely on location and volumes of the nodes; no image intensity is taken into account. Here we present the summary of the distribution features and their definitions. The points within primary tumor and lymph nodes were defined based on the clinical contours and 1mm cubic grid. The clinical lymph node contour was additionally split into subcontours for single nodes (Figure 1). The distances between structures are defined as the distances between respective centers of the mass. The following features were then calculated.

I. Features based on the center of the mass location

- Center of mass shift between primary tumor contour and contour encompassing primary tumor and lymph nodes, CM – center of mass.

  1)

  $$\text{center of mass shift} = \sqrt{(CM_{PT} - CM_{PT+LN})^2}$$

- Distribution of distances between primary tumor and lymph nodes, d_{iPT} – distance between the *ith* lymph node and primary tumor, *n* – number of nodes, d_{ij} – distance between the *ith* and *jth* lymph node and primary tumor.

  2)

  $$\text{largest distance} = \max(d_{iPT})$$

  3)

  $$\text{mean distance} = \frac{1}{n}\sum_{i=1}^{n} d_{iPT}$$

  4)

  $$\text{sum of distances} = \sum_{i=1}^{n} d_{iPT}$$

  5) sum of distances between primary tumor and lymph nodes distances normalized by the average distance between lymph nodes.

  $$\text{normalized sum of distances} = \frac{\sum_{i=n}^{n} d_{iPT}}{\frac{1}{n}\sum_{i=1}^{n}\sum_{j=i+1}^{n} d_{ij}}$$

- Distribution of distances between primary tumor and lymph nodes weighted by lymph node's volume, v_i – volume of the *ith* lymph node.

  6)

  $$\text{largest weighted distance} = \max(d_{iPT} v_i)$$

  7)

  $$\text{mean weighted distance} = \frac{1}{n} \sum_{i=1}^{n} d_{iPT} v_i$$

- Distribution of distances between primary tumor and lymph nodes weighted by lymph node's volume and normalized by tumor volume, v_{PT} – volume of primary tumor.

  8)

  $$\text{normalized largest weighted distance} = \max\left(\frac{d_{iPT} v_i}{v_{PT}}\right)$$

  9)

  $$\text{normalized mean weighted distance} = \frac{1}{n} \sum_{i=1}^{n} \frac{d_{iPT} v_i}{v_{PT}}$$

- Distribution of the smallest distances between the analyzed structures based on Kruskal algorithm [1] (see Figure 2), $mind_i$ – distribution of minimal distances, μ_{mind} – mean of the distribution of minimal distances.

  10) mean of the distribution of minimal distances

  $$\text{distance distribution mean} = \frac{1}{(n+1)} \sum_{k=1}^{n+1} mind_k$$

  11) variance of the distribution of minimal distances

  $$\text{distance distribution variance} = \frac{1}{(n+1)} \sum_{k=1}^{n+1} (mind_k - \mu_{mind})^2$$

II. Features based on all the points within contours

- Optimal number of clusters in kmeans clustering based on Calinski-Harabasz index and points within lymph nodes contour [2].

  12)

  optimal cluster number

- Principal component analysis based on all the points within primary tumor and lymph nodes [3].

  13)

  $$\text{elongation} = \sqrt{\frac{minor\ principal\ axis}{major\ principal\ axis}}$$

  14)

  $$\text{flatness} = \sqrt{\frac{least\ principal\ axis}{major\ principal\ axis}}$$

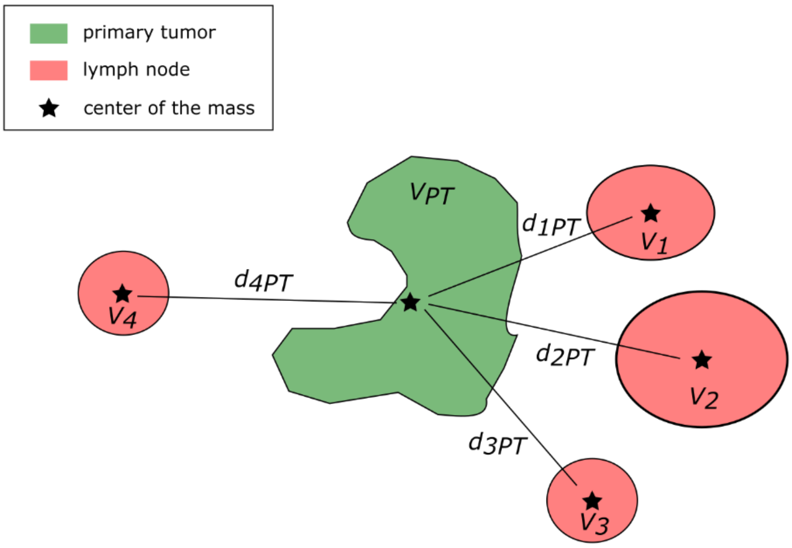

Figure 1. Scheme of the lymph nodes distribution around primary tumor; v*i* – volume of *ith* lymph node, v_{PT} – volume of primary tumor, d_{iPT} – distance between center of the mass of primary tumor and *ith* lymph node

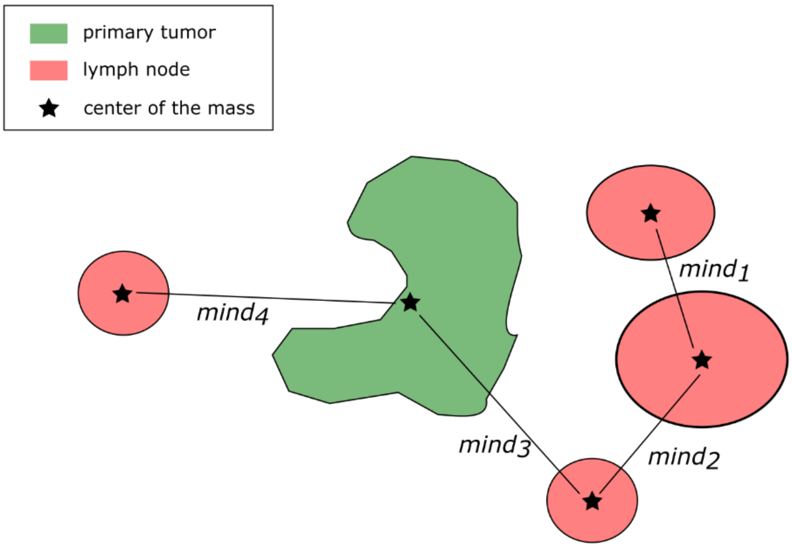

Figure 2. Scheme of the lymph nodes distribution around primary tumor additionally showing the shortest path between the structures

Literature

1. Cormen TH, Leiserson CE, Rivest RL, Stein C: **Introduction to algorithms**: MIT press; 2009.
2. Caliński T, Harabasz J: **A dendrite method for cluster analysis**. *Communications in Statistics-theory and Methods* 1974, **3**(1):1-27.
3. Zwanenburg A, Leger S, Vallières M, Löck S, for the Image Biomarker Standardisation Initiative: **Image biomarker standardisation initiative**. *arXiv:161207003v4* 2017.
